# A New Paradigm of Cardio-Hematological Monitoring in Chronic Myeloid Leukemia Patients Treated With Tyrosine Kinase Inhibitors

**DOI:** 10.7759/cureus.25766

**Published:** 2022-06-08

**Authors:** Nataliia Lopina, Iryna Dmytrenko, Dmytro Hamov, Dmytro Lopin, Iryna Dyagil

**Affiliations:** 1 ClinCaseQuest, Med Inform Group LLC, Kharkiv, UKR; 2 Department of Radiation Oncohematology and Stem Cell Transplantation, National Research Center for Radiation Medicine of National Academy of Medical Sciences of Ukraine, Kyiv, UKR; 3 Regional Medical Hematology Center, Cherkasy Regional Oncology Dispensary, Cherkasy Regional Council, Cherkassy, UKR; 4 Department of Ultrasound Diagnostic and Minimally Invasive Interventions, SI Zaitsev V.T. Institute of General and Urgent Surgery of National Academy of Medical Science of Ukraine, Kharkiv, UKR

**Keywords:** statins, cardiovascular prevention strategy for cardiovascular events, cardiovascular risk, cardiovascular diseases, tkis, tyrosine kinase inhibitors, chronic myeloid leukemia

## Abstract

Significant progress has been achieved in treating patients with onco-hematological diseases, including chronic myeloid leukemia (CML). This is primarily associated with the development of targeted therapy involving tyrosine kinase inhibitors (TKIs), such as imatinib, nilotinib, bosutinib, dasatinib, and ponatinib. Along with the increased survival of patients with CML, special attention has recently been paid to cardiovascular complications in CML patients due to the prevalence of cardiovascular diseases in the general population and the toxicity profile of targeted drugs.

This article presents the strategy for reducing cardiovascular risk in CML patients treated with TKIs. We discuss the components of cardiovascular risk in CML patients and the findings of current studies. Current data confirm the increased cardiovascular risk in the CML population compared to the general population, which necessitates the widespread introduction of cardiovascular prevention strategies in CML patients. The pharmacokinetics and pharmacodynamics of TKIs on the cardiovascular system are discussed. We propose two main approaches in the strategy of cardiovascular risk prevention in patients with CML, namely, before the start of TKI administration and during TKI treatment. This article presents the diagnostic assessment before prescribing TKIs, as well as while monitoring TKI therapy, and discusses the features of the choice of TKIs depending on patients’ general and cardiovascular comorbidity. Emphasis is placed on the risk stratification in patients with CML following general population algorithms, lifestyle modifications, and statin therapy for achieving the target levels of cardiovascular indicators. We also discuss unsolved questions in the current clinical guidelines and ways to further develop a cardiovascular risk-reducing strategy for CML patients.

## Introduction and background

Following the introduction of tyrosine kinase inhibitors (TKIs) in clinical practice in 2001, chronic myeloid leukemia (CML) has transformed from a potentially fatal disease into a controllable disease [1]. The life expectancy of patients with CML has significantly increased and is approaching the general population level [1]. Given that the leading cause of death in the general population is cardiovascular disease (CVD), the issue of reducing cardiovascular risk is becoming relevant for patients with CML based on increased life expectancy in CML patients and the cardiovascular toxicity of TKIs. This problem is now attracting increasing attention from onco-hematologists and cardiologists around the world to improve the prognosis and life expectancy in this group of patients [2-4].

Cardiovascular risk in the CML population is higher than that in the general population [5]. Many studies have demonstrated the cardiotoxicity of TKIs. Cardiotoxicity differed depending on the type of TKIs [3,5-14].

The increased risk of CVD in patients with CML treated with TKIs consists of several components. Similar to the general population patients with CML have traditional cardiovascular risk factors and concomitant CVDs that can precede the development of CML. In addition to the combination of a basic state of the cardiovascular system, CML patients have the additional cardiotoxic action of TKIs [3,5-14].

Cardiovascular toxicity induced by TKIs has complex pathogenesis and the pathophysiologic mechanisms that affect the cardiovascular system remain unclear. Lesions of the cardiovascular system develop with TKI administration due to the effect of these drugs on vessels, metabolism, and myocardium [15-20].

The strategy for reducing cardiovascular risk in patients with CML treated with TKIs should be based on the individual patient risk assessment, general population recommendations [21], and cardiotoxic features of the TKI used for CML treatment. All CML patients should be recommended a two-stage approach for reducing cardiovascular risk. The first stage is risk assessment and the choice of appropriate TKIs before starting therapy and prevention of cardiovascular complications. The second stage is monitoring and treatment during TKI therapy with further prevention of cardiovascular complications.

Statins are used for primary and secondary cardiovascular prevention in the general population. According to some guidelines, there is a need to prescribe statins to patients with CML; however, there are numerous unresolved questions [3].

Recent research data have confirmed the benefits of the concomitant use of statins and TKIs, both for reducing cardiovascular risks and for achieving deep molecular response for CML [22,23].

In this review, we propose a decision-making algorithm for starting hypolipidemic therapy in CML patients, which can answer the questions of when and with whom to start prophylaxis, as well as how intensive lipid-lowering therapy should be.

## Review

Cardiovascular risk in patients with CML

Cardiovascular risk in the CML population is higher than that in the general population. In 2017, a population-based study evaluating the prevalence of CVD and CVD risk factors in 1,639 CML patients in the United States was published. At a five-year follow-up, the prevalence of CVD conditions and CVD risk factors was 33.0% and 77.7%, respectively. Compared with the general US adult population, the standardized prevalence rates at one year in patients with CML were significantly higher by factors of 1.3-3.5 times for CVD conditions and 20% to 40% significantly higher for hypertension, diabetes, and obesity (p < 0.001) [5]. Patients with CML treated with some of the TKIs have an increased cardiovascular risk based on randomized controlled trials. An increased number of cardiovascular events in CML patients treated with nilotinib as the first-line was demonstrated in the five-year follow-up of the ENESTnd clinical trial (nilotinib vs. imatinib) [6,7].

There was also an increase in the incidence of peripheral artery disease, as well as biochemical and ankle-brachial index changes in patients taking nilotinib. The increase in the incidence of peripheral arterial disease was accompanied by the necessity for surgical interventions, both minimally invasive (stenting) and major surgical interventions (amputation of the lower extremities) [6,7].

Although this study was not originally intended to assess cardiovascular safety, it was the first time that a higher incidence of vascular events was demonstrated in patients receiving nilotinib compared to imatinib. During the study, cardiovascular events, namely, ischemic heart disease, ischemic cerebrovascular events, and/or peripheral artery disease, occurred most frequently among patients in the high cardiovascular risk group (nilotinib 300 mg twice daily, 17.5%; nilotinib 400 mg twice daily, 23.7%; imatinib, 3.0%) and intermediate-risk group (12.2%, 25.0%, and 4.1%, respectively) in each arm, whereas patients in the low-risk category in each arm experienced fewer cardiovascular events by the data cutoff (1.7%, 6.3%, and 1.1%, respectively). These data suggest that the toxicity associated with nilotinib affects all arterial channels [7].

Substantial vascular toxicity was later demonstrated in studies with ponatinib. After 12 months of follow-up in the PACE study, 6% of patients had coronary events, 3% had cerebrovascular events, and 4% had peripheral vascular events. The level of events increased to 10%, 7%, and 7%, respectively, on 28 months of follow-up. A retrospective analysis of the PACE database showed a higher risk of vascular toxicity in patients with pre-existing cardiovascular risk factors or pre-existing CVD and was a predictor of dose-dependent cardiovascular events. In addition, at least a quarter of patients developed hypertension after starting treatment with ponatinib. Aggregate data convincingly suggest that ponatinib is associated with a higher risk of cardiovascular side effects compared to other TKIs [8,9].

The cardiovascular risk profile of ponatinib has drawn attention to the vascular and metabolic effects of other TKIs used in the treatment of CML. These risks should be considered before choosing TKIs for CML treatment. For example, a small percentage of patients receiving both dasatinib and nilotinib experienced QT prolongation. No cases of ventricular arrhythmia were reported with QT prolongation with these drugs [6,7]. For this reason, QT assessment using an electrocardiogram is recommended when prescribing nilotinib [2-4]. In the case of dasatinib, dyspnea observed in some patients prompted the Food and Drug Administration (FDA) to issue warnings and recommend that patients be screened for signs and symptoms of cardiopulmonary disease before and during treatment. A significant proportion of patients had pleural and pericardial effusions in initial studies with dasatinib. Moreover, reports of pulmonary hypertension have subsequently been reported with dasatinib [3]. In 2012, the French Pulmonary Hypertension Registry reported nine severe cases of pulmonary hypertension associated with dasatinib [3,10,11]. During the initial diagnosis, patients with CML had precapillary pulmonary hypertension in moderate and severe forms with severe symptoms. The incidence of dasatinib-associated pulmonary hypertension based on randomized trials is 3%. However, in these trials, patients did not undergo a systematic examination for pulmonary hypertension before [3,10-12]. The DASISION five-year clinical trial report (dasatinib vs. imatinib in untreated patients with CML, where dasatinib was compared to first-line imatinib) noted a 5% risk of developing ischemic arterial events in patients with a 2% risk of imatinib [12].

CML patients treated with TKIs developed vascular events, including cardiac, cerebral, and peripheral, more frequently than the general population of the same age. Nilotinib and ponatinib were associated with vascular complications.

Relatively recently, the results of the first studies comparing the usage of imatinib 400 mg, imatinib 800 mg, nilotinib, dasatinib, and ponatinib were published. Ponatinib 45 mg therapy was shown to be associated with an increase in cardiovascular adverse events (4.62; 95% confidence interval (CI) = 2.7-7.7; p < 0.0001) and atherothrombotic adverse events (6.38; 95% CI = 1.8-21.8; p < 0.0001) compared with imatinib 400 mg [13]. The same study demonstrated an increasing trend in cardiovascular risk in patients with CML treated with TKIs, mostly during the first year of treatment. New adverse atherothrombotic complications were the highest in the first year after TKI initiation than registered in subsequent years.

Hence, the relevance of cardiovascular monitoring is important not only in the long-term prognosis of patients with CML but also for short-term prognosis, which should be taken into account before prescribing TKIs.

Another population-based study found that new-generation TKIs are more likely to develop cardiovascular events in CML patients than in imatinib [14]. This retrospective population-based cohort study included patients receiving first-line imatinib, dasatinib, and nilotinib between January 1, 2007, and December 31, 2016 (1,207 patients were included). Athero-thromboembolic events were the primary outcome, while other events related to the cardiovascular system were secondary outcomes. Patients receiving nilotinib had a significantly higher risk of atherothrombotic events (hazard ratio (HR) = 4.92, 95% CI = 1.68-14.36) than those receiving imatinib. Conversely, no difference was found for other cardiovascular events. The risks of atherothrombotic events and other cardiovascular events were similar between dasatinib and imatinib and between nilotinib and dasatinib. Patients receiving nilotinib had a significantly higher risk of atherothrombotic events than patients receiving imatinib. However, the risk of atherothrombotic events and other cardiovascular events did not differ significantly between dasatinib and imatinib [14].

Components of cardiovascular risk in patients with CML treated with TKIs

**Figure 1:**
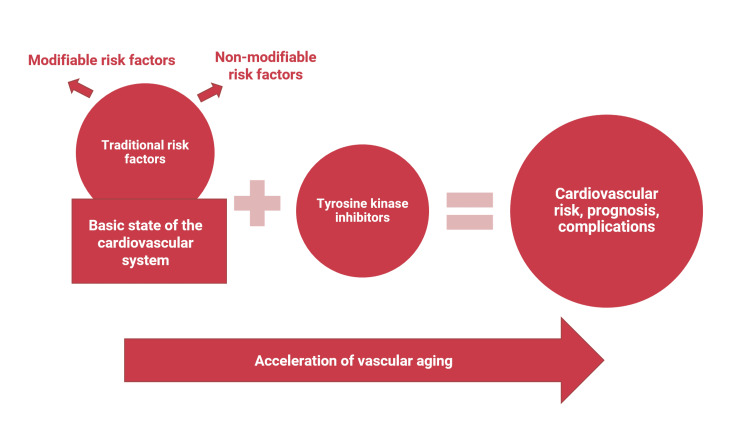
Components of cardiovascular risk in patients with CML treated with TKIs (in accordance with the opinion of the authors of the article based on clinical trials and review publications). Image credits: Nataliia Lopina, Iryna Dmytrenko, Dmytro Hamov, Dmytro Lopin, and Iryna Dyagil. CML: chronic myeloid leukemia; TKIs: tyrosine kinase inhibitors

The increased risk of cardiovascular disease in patients with CML treated with TKIs consists of several components. In the general population, patients with CML have traditional cardiovascular risk factors and concomitant CVDs that can precede the development of CML. In addition to the combination of a basic state of the cardiovascular system with cardiovascular risk factors, CML patients have the additional action of TKIs. Traditional cardiovascular risk factors, the basic state of the cardiovascular system, or existing concomitant CVDs accelerate the process of vascular aging, form the cardiovascular risk of a particular patient, and determine the prognosis for overall survival (Figure 1).

Traditional risk factors include modifiable and non-modifiable risk factors. Modifiable risk factors can be changed by lifestyle alterations or medications. Non-modifiable risk factors cannot be controlled by patients (Table 1).

**Table 1:** Risk factors for cardiovascular disease in the general population. CAD: coronary artery disease; LDL-C: low-density lipoprotein cholesterol; HDL-C: high-density lipoprotein cholesterol; TG: triglycerides

Modifiable risk factors	Non-modifiable risk factors
Smoking	Individual history of CAD
Dyslipidemia (LDL-C level increase, HDL-C level decrease, TG level increase)	Family history of CAD
Increased blood pressure	Age
Diabetes	Sex
Obesity	
Dietary factors	
Low physical activity	
Alcohol	

The Framingham Heart Study, which examined the risk factors for CVD and assessed the risk of developing coronary artery disease, found that a combination of the above-mentioned risk factors increased the risk of coronary artery disease [15].

Pathophysiologic effect of TKIs on the cardiovascular system

Cardiovascular toxicity induced by TKIs has complex pathogenesis, and the pathophysiology mechanisms that affect the cardiovascular system remain unclear. It is generally accepted that various factors can play an important role in this process.

Lesions of the cardiovascular system develop with TKI administration due to the effect of these drugs on vessels, metabolism, and myocardium.

TKIs can cause endothelial dysfunction, which is the basis for the development of atherosclerosis. Some TKIs, in particular nilotinib, have been shown to increase the production of proatherogenic surface molecules in endothelial cells [16]. Studies also suggest that nilotinib often raises glucose levels and sometimes causes or exacerbates pre-existing type 2 diabetes mellitus by blocking the post-insulin receptor. ABL tyrosine kinase is involved in the transmission of the insulin pathway, and its blocking by TKIs may result in decreased insulin sensitivity and the need for excess insulin secretion. Moreover, exposure to nilotinib also appears to reduce adiponectin levels, thereby impairing tissue sensitivity to insulin [17].

Hypertension is a common side effect of nilotinib and ponatinib treatment. Both of these drugs strongly inhibit vascular endothelial growth factor (VEGF). VEGF physiologically causes vasodilation in adult models of peripheral vascular disease and myocardial ischemia through the acute release of nitric oxide (NO). VEGF activates arterial vasodilation dependent on NO and regulates the production of NO, which acts on endothelial cells, thus regulating basal arterial tone and blood pressure. VEGF and VEGF receptor inhibitors block this mechanism and may cause hypertension. Hypertension itself may be a potential risk factor for other and more severe cardiovascular events, such as myocardial infarction and stroke [18]. VEGF also plays an important role in the kidneys in cell proliferation and homeostasis. Inhibition of its pathway can lead to glomerular dysfunction, identifying or exacerbating existing proteinuria and hypertension. These cells play an important role in the repair of the vascular wall, producing and releasing heparin and other bioactive tissue activators of plasminogen. Both nilotinib and ponatinib have inhibitory activity and may impair mast cell function. Imatinib has been shown to be unable to impair endothelial cell regeneration and cause endothelial sensitization to progress to atherosclerosis, suggesting that mast cell disruption alone is not sufficient to determine the proatherogenic phenotype in the endothelium [19,20].

Mechanisms involved in the development of cardiotoxicity are based on the inhibition of the main target molecules used to treat CML (inhibition of ABL tyrosine kinase, on-target effects) and other kinases that are not involved in the pathogenesis of the disease (off-target) [16-20].

Moreover, each TKI may have a different spectrum of cardiac and/or vascular toxicity in patients depending on their age, sex, comorbidities, and the presence of additional common cardiovascular risk factors (e.g., smoking habits, dyslipidemia, overweight, lifestyle, diabetes mellitus). Different TKIs have different molecular targets, which also determine their specific effects on the cardiovascular system and the different spectrum of side effects. Table 2 presents the TKIs from the least cardiotoxic (imatinib) to the most cardiotoxic (ponatinib) with their molecular targets and vascular effects [16].

**Table 2:** Tyrosine kinase inhibitors for CML treatment (molecular target and effects on the cardiovascular system). Adapted from Manouchehri A, Kanu E, Mauro MJ, Aday AW, Lindner JR, Moslehi J: Tyrosine kinase inhibitors in leukemia and cardiovascular events: from mechanism to patient care. Arterioscler Thromb Vasc Biol. 2020, 40:301-8. 10.1161/ATVBAHA.119.313353 [16]. Permission was obtained from the original authors. ABL1: ABL proto-oncogene 1; CAD: coronary artery disease; CML: chronic myeloid leukemia; FGFR: fibroblast growth factor receptor PDGFR: platelet-derived growth factor receptor; VEGFR: vascular endothelial growth factor receptor; VTE: venous thromboembolism

Tyrosine kinase inhibitor	Year of implementation	Molecular target	Vascular effect
Imatinib	2001	PDGFR-α (0.1), KIT (0.1), ABL1 (0.6)	Positive metabolic profile for blood glucose and lipid profile
Bosutinib	2014	ABL1 (4.4), FGFR 2, VEGFR 2, PDGFRβ, SRC (1.2)	Hypertension
Dasatinib	2006	ABL1 (0.27), KIT (79.0), PDGFR-α, PDGFR-β, SRC (0.8)	Platelet dysfunction, pulmonary hypertension, pleural effusion
Nilotinib	2007	ABL1 (18.5), KIT, PDGFR-α	Peripheral artery disease, CAD, hyperglycemia, dyslipidemia, QT prolongation
Ponatinib	2012	ABL1 (3.7), FGFR 1 (2.2), FGFR 2 and 3, VEGFR 1, 2, and 3, PDGFR-α (1.1), PDGFR-β, KIT, SRC (5.4), TIE2	Peripheral artery disease, hypertension, CAD, VTE, hyperglycemia

Reduction of cardiovascular risk in patients with CML treated with TKIs

Clinicians should focus all efforts on the treatment of CML patients to achieve large molecular response, deep molecular response, and treatment-free remission (TFR) [3,4,11].

At the same time, сlinicians should not forget about the necessity of the prevention strategy to reduce cardiovascular complications and metabolic disorders during TKI treatment in patients with CML, such as coronary artery disease, acute myocardial infarction, diseases of peripheral arteries, dyslipidemia, diabetes mellitus, and carbohydrate intolerance.

The fact that cardiovascular adverse events occur in CML patients during TKI therapy at current (or future) cardiovascular risk confirms the hypothesis that primary prevention is a key strategy in reducing the risk of cardiovascular events in these patients. The 2020 ELN Guidelines for the Diagnosis and Treatment of CML report cardiovascular adverse events as major non-hematological adverse reactions and strongly recommend that the use of ponatinib and nilotinib should be avoided in patients with pre-existing or concomitant vascular disease [11].

Based on available data we think that there are two key stages in the strategy of reducing cardiovascular risk in CML patients. The first stage is risk assessment and choice of appropriate TKIs before starting therapy and prevention of cardiovascular complications. The second stage is monitoring and treatment during TKI therapy and the prevention of cardiovascular complications.

Before starting and choosing TKI therapy all patients with CML should be provided an assessment of the cardiovascular risk with the definition of the risk category (low, intermediate, high, very high) in accordance with the general population algorithm, electrocardiogram (ECG), determination of baseline values of lipid profile and fasting glucose and/or HbA1c, and assessment of the presence of comorbidities for the optimal choice of TKIs.

**Table 3:** Diagnostic minimum for CML patients at the initial contact before starting therapy with TKIs (emphasis on indicators of cardiovascular risk). Barber MC, Mauro MJ, Moslehi J: Cardiovascular care of patients with chronic myeloid leukemia (CML) on tyrosine kinase inhibitor (TKI) therapy. Hematology Am Soc Hematol Educ Program. 2017, 2017:110-4. 10.1182/asheducation-2017.1.110 [2]. Permission was obtained from the original authors. CML: chronic myeloid leukemia; ECG: electrocardiogram; HbA1c: hemoglobin A1c; TKIs: tyrosine kinase inhibitors

	Imatinib	Bosutinib	Dasatinib	Nilotinib	Ponatinib
Blood pressure control	Recommended	Recommended	Recommended	Recommended	Recommended
Ankle-brachial index	If clinically indicated	If clinically indicated	If clinically indicated	Recommended	Recommended
Basic control of metabolic parameters	Recommended	Recommended	Recommended	Recommended	Recommended
Lipid profile	Recommended	Recommended	Recommended	Recommended	Recommended
Fasting glucose/HbA1_C_	Recommended	Recommended	Recommended	Recommended	Recommended
ECG	Recommended	Recommended	Recommended	Recommended	Recommended
Echocardiography	If clinically indicated	If clinically indicated	Recommended	If clinically indicated	Recommended

Algorithm for assigning first-line TKIs based on cardiovascular risk assessment

Physicians should take into account concomitant conditions and diseases while choosing the TKIs for CML therapy. Clinical guidelines recommend choosing TKIs that have no contraindications to their use in each particular case [3,11] (Table 4).

**Table 4:** Recommendations for the selection of first-line TKIs based on the assessment of comorbidities and concomitant diseases. Smith G, Apperley J, Milojkovic D, et al.: A British Society for Haematology Guideline on the diagnosis and management of chronic myeloid leukaemia. Br J Haematol. 2020, 191:171-93. 10.1111/bjh.16971 [3]. Permission was obtained from the publisher, John Wiley and Sons. TKIs: tyrosine kinase inhibitors

Concomitant pathology	Bosutinib	Dasatinib	Imatinib	Nilotinib
Hypertension	No contraindication	No contraindication	No contraindication	Intermediate risk of exacerbation of pre-existing condition
Coronary artery disease	No contraindication	No contraindication	No contraindication	Intermediate risk of exacerbation of pre-existing condition
Cerebrovascular thrombosis	No contraindication	No contraindication	No contraindication	Intermediate risk of exacerbation of pre-existing condition
Peripheral arterial occlusive disease	No contraindication	No contraindication	No contraindication	Intermediate risk of exacerbation of pre-existing condition
Prolonged QT interval	No contraindication	No contraindication	No contraindication	Avoid if possible
Congestive heart failure	No contraindication	intermediate risk of exacerbation of pre-existing condition	intermediate risk of exacerbation of pre-existing condition	Avoid if possible
Diabetes mellitus	No contraindication	No contraindication	No contraindication	Intermediate risk of exacerbation of pre-existing condition
Gastrointestinal bleeding	Low risk of exacerbation of pre-existing condition	Intermediate risk of exacerbation of pre-existing condition	No contraindication	No contraindication
Pulmonary hypertension	No contraindication	Avoid if possible	No contraindication	No contraindication
Chronic pulmonary disease	No contraindication	Intermediate risk of exacerbation of the pre-existing condition	No contraindication	No contraindication
Pancreatitis	No contraindication	No contraindication	No contraindication	Intermediate risk of exacerbation of the pre-existing condition
Abnormal liver function	Intermediate risk of exacerbation of the pre-existing condition	No contraindication	Low risk of exacerbation of the pre-existing condition	Intermediate risk of exacerbation of the pre-existing condition

In the presence of certain basic cardiovascular diseases, the administration of TKIs, which may worsen the course of these diseases and their progression, should be avoided. Before prescribing TKIs, it is necessary to perform screening for symptoms of coronary artery disease, peripheral artery disease, and pulmonary hypertension. These include mandatory consultation with a cardiologist and providing the assessment of the ankle-brachial index, ultrasound examination of the main vessels, and echocardiography.

The next step before prescribing TKIs should be to determine the cardiovascular risk of the patient following the general population algorithm [21].

In 2021, new risk assessment scales were proposed: SCORE2 (Systematic COronary Risk Evaluation 2) and SCORE2-OP (Systematic COronary Risk Evaluation Older Persons) for almost healthy people according to age. SCORE (Systematic COronary Risk Evaluation) scale was used early. For the stratification of patients, physicians can use special tables of risk in the presence of cardiovascular disease (Table 5) [21].

**Table 5:** Categories and criteria of cardiovascular risk in the general population. Visseren FL, Mach F, Smulders YM, et al.: 2021 ESC Guidelines on cardiovascular disease prevention in clinical practice. Eur Heart J. 2021, 42:3227-337. 10.1093/eurheartj/ehab484 [21]. Permission was obtained from the authors. ASCVD: atherosclerotic cardiovascular disease; AMI: acute myocardial infarction; ACS: acute coronary syndromes; ACR: albumin-to-creatinine ratio: (to convert mg/g to mg/mmol: divide by 10); CKD: chronic kidney disease; CTA: computed tomography angiography; DM: diabetes mellitus; eGFR: estimated glomerular filtration rate; TIA: transient ischaemic attack; TOD: target organ damage; PAD: peripheral artery disease; SCORE: Systematic COronary Risk Evaluation; SCORE2: Systematic COronary Risk Evaluation 2; SCORE2-OP: Systematic COronary Risk Evaluation Older Persons

Cardiovascular risk group	Criteria
Very high risk	Documented ASCVD, clinical or unequivocal on imaging. Documented clinical ASCVD includes previous AMI, ACS, coronary revascularization and other arterial revascularization procedures, stroke and TIA, aortic aneurysm, and PAD. Unequivocally documented ASCVD on imaging includes plaque on coronary angiography, carotid ultrasound, or CTA. It does NOT include some increase in continuous imaging parameters such as intima-media thickness of the carotid artery
	Familial hypercholesterolemia with confirmed CAD of the atherosclerotic origin or another major risk factor
	Severe chronic kidney disease without diabetes or ASCVD (eGFR<30 mL/minute/1.73 m^2^ or eGFR 30−44 mL/minute/1.73 m^2^ and ACR >30)
	SCORE score ≥10%
	Score on SCORE2 and SCORE2-OP (<50 years: ≥7.5%; 50–69 years: ≥10%; ≥70 years: ≥15%)
	Patients with DM with established ASCVD and/or severe TOD: eGFR <45 mL/minute/1.73 m^2^ irrespective of albuminuria; eGFR 45–59 mL/min/1.73 m^2^ and microalbuminuria (ACR 30–300 mg/g); Proteinuria (ACR >300 mg/g); presence of microvascular disease in at least three different sites (e.g., microalbuminuria plus retinopathy plus neuropathy)
High risk	Significant increase in one of these risk factors (total cholesterol ˃8 mmol/l or LDL cholesterol ˃4.9 mmol/L or blood pressure ≥180/110 mmHg)
	Familial hypercholesterolemia without additional risk factors
	Diabetes mellitus without TOD or diabetes mellitus lasting ˃10 years or another additional risk factor.
	Moderate CKD without diabetes or ASCVD (eGFR 30−44 mL/minute/1.73 m^2^ and ACR <30 or eGFR 45−59 mL/minute/1.73 m^2^ and ACR 30−300 or eGFR ≥60 mL/minute/1.73 m2 and ACR >300)
	Patients with DM without ASCVD and/or severe TOD, and not fulfilling the moderate risk criteria
	SCORE score 5-9%
	Score on SCORE2 and SCORE2-OP (<50 years: 2.5 to <7.5%; 50–69 years: 5 to <10%; ≥70 years: 7.5 to <15%)
Intermediate risk	Young patients (for type 1 diabetes <35 years; for type 2 diabetes <50 years) with diabetes <10 years without other additional risk factors
	Patients with well-controlled short-standing DM (e.g., <10 years), no evidence of TOD, and no additional ASCVD risk factors
	SCORE score 1–4%
	Score on SCORE2 and SCORE2-OP (<50 years: <2.5%; 50–69 years: <5%, ≥70 years: <7.5%)
Low risk	SCORE score <1%
	Score on SCORE2 and SCORE2-OP (<50 years: <2.5%; 50–69 years: <5%, ≥70 years: <7.5%)

Patients with CML at high and very high cardiovascular risk should not be administered drugs with a high cardiotoxicity profile, and it is recommended to prescribe alternative TKIs with a low risk of cardiovascular events [3,11]. Risk assessment plays a significant role not only in choosing appropriate TKIs but also in developing a prevention strategy and frequency of monitoring for each patient. Patient assessment should be performed by a multidisciplinary team of specialists with a discussion of the main purpose of treatment by onco-hematologists and cardiologists.

Algorithms for managing patients during TKI therapy

Modification of cardiovascular risk factors and lifestyle should be recommended for all patients with CML. Patients should be recommended simple practical ABCDE steps to reduce the risk of coronary artery disease in patients with CML treated with TKIs, as shown in Table 6 [2].

**Table 6:** Practical ABCDE steps to reduce cardiovascular risk in patients with CML receiving therapy with TKIs. CML: chronic myeloid leukemia; ECG: electrocardiogram; TKIs: tyrosine kinase inhibitors

ABCDE prevention steps	Actions
А	Awareness of cardiovascular disease signs and symptoms
	Aspirin (in selected patients)
	Ankle-brachial index measurement at baseline and follow-up to document peripheral arterial disease
В	Blood pressure control
С	Cigarette and tobacco cessation
	Cholesterol control (regular monitoring and treatment, if treatment indicated)
D	Diabetes mellitus (regular monitoring and treatment, if treatment indicated)
	Diet and weight management
E	Exercise
	ECG
	Echocardiography

It is recommended to monitor the following cardiovascular risk factors: fasting blood pressure and fasting glucose/HbA1C one month after starting therapy, as shown in Table 7 [2,3].

**Table 7:** Cardiovascular diagnostic management after the first month of TKI treatment. Barber MC, Mauro MJ, Moslehi J: Cardiovascular care of patients with chronic myeloid leukemia (CML) on tyrosine kinase inhibitor (TKI) therapy. Hematology Am Soc Hematol Educ Program. 2017, 2017:110-4. 10.1182/asheducation-2017.1.110 [2]. Permission was obtained from the original authors. HbA1c: hemoglobin A1c; TKI: tyrosine kinase inhibitor

	Imatinib	Bosutinib	Dasatinib	Nilotinib	Ponatinib
Blood pressure control	Recommended	Recommended	Recommended	Recommended	Recommended
Fasting glucose/HbA1C	If clinically indicated	If clinically indicated	If clinically indicated	Recommended	Recommended

**Figure 2:**
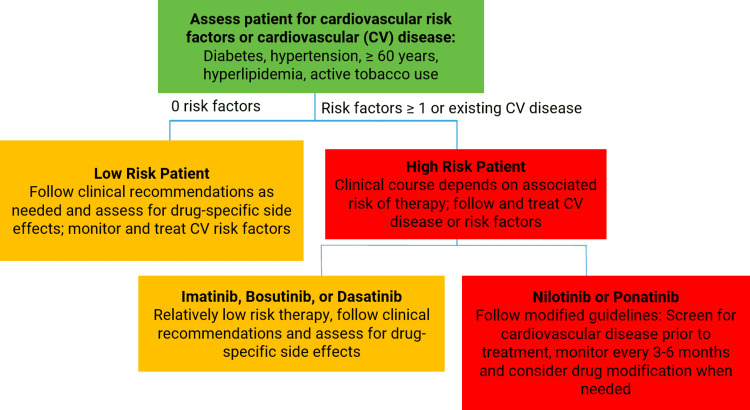
Strategy for monitoring patients with CML depending on the cardiovascular risk and the drug of targeted therapy. Barber MC, Mauro MJ, Moslehi J: Cardiovascular care of patients with chronic myeloid leukemia (CML) on tyrosine kinase inhibitor (TKI) therapy. Hematology Am Soc Hematol Educ Program. 2017, 2017:110-4. 10.1182/asheducation-2017.1.110 [2]. Permission was obtained from the original authors. CML: chronic myeloid leukemia; CV: cardiovascular

During TKI therapy, the frequency of monitoring the state of the cardiovascular system may depend on the cardiovascular risk of the patient and the drug selected for CML treatment [2] (Figure 2, Table 8). Cardiovascular markers should be evaluated in patients receiving TKIs every three or six months, as shown in Table 8 [2,3].

**Table 8:** Frequency of cardiovascular monitoring in patients with CML during TKI therapy (every three or six months). Barber MC, Mauro MJ, Moslehi J: Cardiovascular care of patients with chronic myeloid leukemia (CML) on tyrosine kinase inhibitor (TKI) therapy. Hematology Am Soc Hematol Educ Program. 2017, 2017:110-4. 10.1182/asheducation-2017.1.110 [2]. Permission was obtained from the original authors. CML: chronic myeloid leukemia; ECG: electrocardiogram; TKIs: tyrosine kinase inhibitors

	Imatinib	Bosutinib	Dasatinib	Nilotinib	Ponatinib
Blood pressure control	Recommended	Recommended	Recommended	Recommended	Recommended
Ankle-brachial index	If clinically indicated	If clinically indicated	If clinically indicated	Recommended	Recommended
Lipid profile	If clinically indicated	If clinically indicated	If clinically indicated	Recommended	Recommended
Fasting glucose/HbA1C	If clinically indicated	If clinically indicated	If clinically indicated	Recommended	Recommended
ECG	If clinically indicated	If clinically indicated	Recommended	Recommended	Recommended
Echocardiography	If clinically indicated	If clinically indicated	If clinically indicated	If clinically indicated	If clinically indicated

In patients taking nilotinib and ponatinib, it is mandatory to perform the determination of ankle-brachial index, as well as Doppler Ultrasound examination for timely detection of peripheral arterial disease, regardless of cardiovascular risk [2,3]. Patients treated with dasatinib who have a potential risk of pulmonary hypertension development, as well as any cardiopulmonary symptoms, should undergo regular ECG and echocardiography [2,3].

Target levels of blood pressure, lipids, and glycemia, listed in Table 9 [21], can be used in patients with CML to control cardiovascular risks for primary prevention as in the general population.

**Table 9:** Target levels of blood pressure, lipids, and glycemia in the primary cardiovascular prevention strategy. Visseren FL, Mach F, Smulders YM, et al.: 2021 ESC Guidelines on cardiovascular disease prevention in clinical practice. Eur Heart J. 2021, 42:3227-337. 10.1093/eurheartj/ehab484 [21]. Permission was obtained from the authors. HbA1c: hemoglobin A1c; HDL-cholesterol: high-density lipoprotein cholesterol; LDL-cholesterol: low-density lipoprotein cholesterol; non-HDL cholesterol: non-high-density lipoprotein cholesterol

Risk factor	Target levels
Diabetes	
HbA1c, %	≤7
Lipids	
Total cholesterol (mmol/L)	<4.0
HDL-cholesterol (mmol/L)	≥1.0
LDL-cholesterol (mmol/L)	<2.6 (primary target), <1.8 (at high risk), <1.4 (at very high risk)
non-HDL cholesterol (mmol/L)	<2.5
Triglycerides (mmol/L)	<2.0
Blood pressure (mmHg)	<140/90, ≤130/80 if well tolerated

Also, regardless of the risk, it is necessary to consider statin administration. In patients at high and very high cardiovascular risk, statin therapy should be more aggressive. In the general population, the recommended LDL-cholesterol target level in patients with very high cardiovascular risk is less than 1.4 mmol/L or a decrease of more than 50% from baseline, at high risk the target level is less than 1.8 mmol/L or a decrease of more than 50% from baseline, at intermediate risk the target level is less than 2.6 mmol/L, and at low risk it is less than 3.0 mmol/L [21]. What should be the target level of LDL-cholesterol in CML patients with high and very high cardiovascular risk? This question remains open and clinical guidelines do not provide a clear answer, but it is likely to be justified in this group of patients to achieve target levels of LDL-cholesterol of less than 2.0 mmol/L given the higher cardiovascular risk of cardiovascular complications in this group of patients compared with the general population.

In accordance with the recommendation of the British Society of Hematologists on the diagnosis and management of CML patients (key concepts related to cardiovascular prevention) [3], if the cardiovascular risk is high, very high >10% (according to the SCORE score scale), the use of atorvastatin 20 mg daily should be suggested. Aspirin should not be used for primary prevention in asymptomatic patients, except in patients with carotid artery stenosis of >50%. Blood pressure (BP) should be measured before and during TKI treatment, and hypertension should be treated by a general practitioner following current guidelines. Hypertension is common (>10%) when prescribed in some TKIs, but the risk of life-threatening cardiovascular events is low. TKIs should be stopped if blood pressure is above 180/110 mmHg. QT prolongation is rare: ECG should be performed at the beginning of treatment for patients starting bosutinib and nilotinib or as clinically indicated. In patients with a QT interval estimated by Fridericia (QTcF) of >450 ms (men) or >460 ms (women), electrolyte levels should be measured and further consultation with an appropriate specialist should be provided.

Statins and the achievement of deep molecular response and molecular targets in patients with CML

Inhibitors of HMG-CoA reductase are drugs that have proven effective in preventing cardiovascular events with a level of evidence I (A) (according to RCTs and meta-analyses). Statins should be considered for CML patients for cardiovascular risk reduction.

In recent years, statins have been increasingly prescribed to CML patients receiving second-generation TKI therapy to modify cardiovascular risk factors such as hyperlipidemia. General practitioners may have questions about the safety of combining statins with targeted therapies in patients with CML. Experimental studies have shown that lovastatin synergistically enhances the antileukemic activity of imatinib in cell lines and primary CD34+ cells of patients with CML at different stages of the disease, including patients resistant to imatinib without detected mutations [22]. An international study recently evaluated the effectiveness of a combination of statins and TKIs in the treatment of patients with CML. In a group of 408 patients with CML, the incidence of major molecular response (p = 0.0048) and deep molecular response (p = 0.0016) was significantly higher in patients receiving the statin/TKI combination than in patients receiving TKI alone. The frequency of deep molecular response after five years of therapy was 55.8% (43.4-66.5%) against 41.0% (35.0-47.0%); the frequency of large molecular response after three years of therapy was 77.3% (65.9-85.3%) versus 62.5% (56.7-67.9%), respectively [23].

In addition, in vitro studies have shown that statins synergistically enhance the cytotoxic activity of TKIs against BCR-ABL1+ cell lines (wild-type K562 cell line and K562 cell line with the *T315I *mutation, as well as BaF3 cell lines with *T315*, *G250E*, and *F317L *mutations). A decrease in the colony-forming capacity of murine cKit + lineage − Sca1 + (KLS) cells isolated from CML mice treated with TKIs and statins has been shown in vitro. This suggests that statins can potentially inhibit/destroy leukemic progenitor cells in CML patients. Transcriptome and target RNA sequencing data in this study support the hypothesis that statins inhibit the c-Myc signaling pathway in CML cells. Thus, the additive increased inhibitory activity of TKI and statins against CML cells may be mediated through blockade of the c-Myc pathway, which can be considered a potential therapeutic target for eradication of leukemic progenitor cells in CML patients. The results of the study confirm the therapeutic benefit of concomitant use of statins in the therapy of CML patients treated with TKI. In particular, this combination may be considered to achieve a deep molecular response in patients with CML who have not achieved a deep molecular response to TKI therapy alone. For these patients, the combination of a statin with TKI can help achieve a deep molecular response and subsequently allow discontinuation of TKI [23].

TKIs metabolism peculiarities and concomitant therapy for cardiovascular risk control

Imatinib, dasatinib, nilotinib, ponatinib, and bosutinib are metabolized by cytochrome P450 enzymes, and concomitant use of drugs that are inhibitors of the cytochrome CYP3A4, CYP3A5 system may affect their therapeutic effects (Table 10) [24].

**Table 10:** Cytochromes involved in the process of the liver tyrosine kinase inhibitors biotransformation. CYP: cytochrome P-450 enzymes

Tyrosine kinase inhibitor	Cytochromes involved in the process of biotransformation in the liver
Imatinib	CYP3A4, CYP3A5
Dasatinib	CYP3A4 (preferably), CYP3A5, UGT
Nilotinib	CYP3A4, CYP2C8
Ponatinib	CYP3A4, (CYP2C8, 2D6,3A5 to a lesser extent)
Bosutinib	CYP3A4

Clinical guidelines emphasize that older patients may receive different therapies and/or receive other concomitant medications that are metabolized by CYP450 enzymes (which may reduce the rate of TKI metabolism and increase their toxicity) and therefore may require more frequent dose reduction or treatment interruptions than in young patients. In addition, the peculiarities of TKI metabolism should be considered when choosing a statin for the prevention of cardiovascular events. The only statins that are not metabolized by the cytochrome CYP3A4, and CYP3A5 enzymes are rosuvastatin, fluvastatin, pitavastatin, and pravastatin, which may be considered in CML patients to prevent cardiovascular events. The British Society for Haematology Guidelines on the diagnosis and management of CML also mention atorvastatin for CML patients [3].

Combination hypolipidemic therapy

Occasionally, combination therapy, including ezetimibe, is required to achieve the risk-adjusted target for LDL-cholesterol levels. Expected levels of LDL-cholesterol reduction using one or another treatment regimen are listed in Table 11.

**Table 11:** Expected reduction in low-density lipoprotein-cholesterol for combination therapy. Visseren FL, Mach F, Smulders YM, et al.: 2021 ESC Guidelines on cardiovascular disease prevention in clinical practice. Eur Heart J. 2021, 42:3227-337. 10.1093/eurheartj/ehab484 [21]. Permission was obtained from the authors. PCSK9: proprotein convertase subtilisin/Kexin type 9

Treatment	The average decrease in LDL-cholesterol
Moderate-intensity statin	~30%
High-intensity statin	~50%
High-intensity statin + ezetimibe	~65%
PCSK9 inhibitor	~60%
PCSK9 inhibitor plus high-intensity statin	~75%
PCSK9 inhibitor plus high-intensity statin plus ezetimibe	~85%

The use of combination therapy to achieve target LDL-cholesterol levels is widely used in the general population, and patients with CML should not be an exception to effectively reduce their increased cardiovascular risk.

Decision-making algorithm for starting hypolipidemic therapy in CML patients

Based on the general population algorithm, targeted levels of LDL-cholesterol and the difference in cardiovascular toxicity profiles in CML patients, we propose to use a special decision-making algorithm for starting hypolipidemic therapy in CML patients, which can be useful in routine clinical practice for the physician’s decision when to start statin therapy and for whom and how intensity statin therapy should be performed.

First, regarding cardiovascular risk assessment, identify low, intermediate, high, and very high cardiovascular risk patients. Then, it is necessary to choose the level of cardiovascular toxicity of TKI: low-risk cardiovascular toxicity TKI (imatinib, bosutinib, dasatinib) or high-risk cardiovascular toxicity TKI (nilotinib, ponatinib). And finally, when comparing cardiovascular risk and toxicity profiles, you can get a recommended list of measures for the prevention of cardiovascular complications (Table 12).

**Table 12:** Decision-making algorithm for starting hypolipidemic therapy in CML patients. Table credits: Nataliia Lopina, Iryna Dmytrenko, Dmytro Hamov, Dmytro Lopin, and Iryna Dyagil. *Low-risk cardiovascular toxicity TKIs: imatinib, bosutinib, dasatinib; **High-risk cardiovascular toxicity TKIs: nilotinib, ponatinib CML: chronic myeloid leukemia

	Low-risk cardiovascular toxicity TKIs*	High-risk cardiovascular toxicity TKIs**
Low cardiovascular risk	Repeated risk stratification in a half of year	Repeated risk stratification in a half of year
	Lifestyle modification	Lifestyle modification
	Diet	Diet
	Physical activity	Physical activity
	Blood pressure control and treatment if needed	Blood pressure control and treatment if needed
	TKI treatment adverse effects monitoring	TKI treatment adverse effects monitoring
		Low doses of statin (target LDL-cholesterol level less than 3.0 mmol/L)
Intermediate cardiovascular risk	Repeated risk stratification in a half of year	Repeated risk stratification in a half of year
	Lifestyle modification	Lifestyle modification
	Diet	Diet
	Physical activity	Physical activity
	Blood pressure control and treatment if needed	Blood pressure control and treatment if needed
	TKI treatment adverse effects monitoring	TKI treatment adverse effects monitoring
	Statin (target LDL-cholesterol level less than 2.6 mmol/L)	Statin (target LDL-cholesterol level less than 2.6 mmol/L)
High cardiovascular risk	Lifestyle modification	Lifestyle modification
	Diet	Diet
	Physical activity	Physical activity
	Blood pressure control and treatment if needed	Blood pressure control and treatment if needed
	TKI treatment adverse effects monitoring	TKI treatment adverse effects monitoring
	High-intensity statin therapy or hypolipidemic combination (target LDL-cholesterol level less than 1.8 mmol/L)	High-intensity statin therapy or hypolipidemic combination (target LDL-cholesterol level less than 1.8 mmol/L)
Very cardiovascular high risk	Lifestyle modification	Lifestyle modification
	Diet	Diet
	Physical activity	Physical activity
	Blood pressure control and treatment if needed	Blood pressure control and treatment if needed
	TKI treatment adverse effects monitoring	TKI treatment adverse effects monitoring
	High-intensity statin therapy or hypolipidemic combination (target LDL-cholesterol level less than 1.4 mmol/L)	High-intensity statin therapy or hypolipidemic combination (target LDL-cholesterol level less than 1.4 mmol/L)

Unresolved issues of clinical guidelines

There is no doubt about the necessity of statin therapy in patients with high and very high cardiovascular risk. The unresolved issues of clinical guidelines include the following: target levels of LDL-cholesterol in patients with CML and the comparability of target levels for CML patients with the general population or the necessity to achieve LDL-cholesterol levels following the recommendations for people at high and very high cardiovascular risk, which statin is more recommended in CML patient population, should statins be prescribed to CML patients with low and moderate cardiovascular risk as adjunctive therapy to accelerate the achievement of a deep molecular response, should statins be prescribed to patients receiving TKIs of low and neutral cardiovascular toxicity and at low and moderate cardiovascular risk, who should prescribe and monitor the therapy (hematologists, cardiologists, or family doctors), how the cardio-hematological team of specialists should be formed.

## Conclusions

Reducing cardiovascular risk is an important part of a general treatment strategy for CML patients that can increase patient survival. For reducing cardiovascular risk in patients with CML treated with TKIs, it is necessary to implement a two-stage strategy for cardiovascular disease prevention. The baseline assessment of cardiovascular risk factors, a baseline assessment of cardiovascular system status, patient risk stratification according to the general population algorithm, and selection of the most appropriate TKI based on available cardiovascular risk should be considered before starting targeted therapy. During TKI therapy, implementation of an individual prevention strategy should be considered by performing regular cardiovascular monitoring, repeated re-stratification of risks factors, control of target therapy and its side effects, monitoring the achievement of target levels of LDL-cholesterol, blood pressure control, and blood glucose assessment as the main factors related to the progression of atherosclerotic vascular lesions. Two-stage strategy for cardiovascular disease prevention can help in improving the prognosis of the CML patient population. Such patients should be managed by a multidisciplinary team of specialists, including a hematologist and cardiologist for improving the survival of patients with CML and reducing the risk of cardiovascular events.

It is also important to promote the reduction of cardiovascular risk in patients with CML among health professionals who are involved in providing medical care to this patient group (family doctors, cardiologists). Patient organizations and teaching patients cardiovascular risk reduction strategies can play a leading role in this process. Cardio-hematological monitoring is the basis of success in the modern strategy of treating patients with CML. The rapid development of cardio-oncology will help improve the provision of highly specialized medical care to patients with CML.
